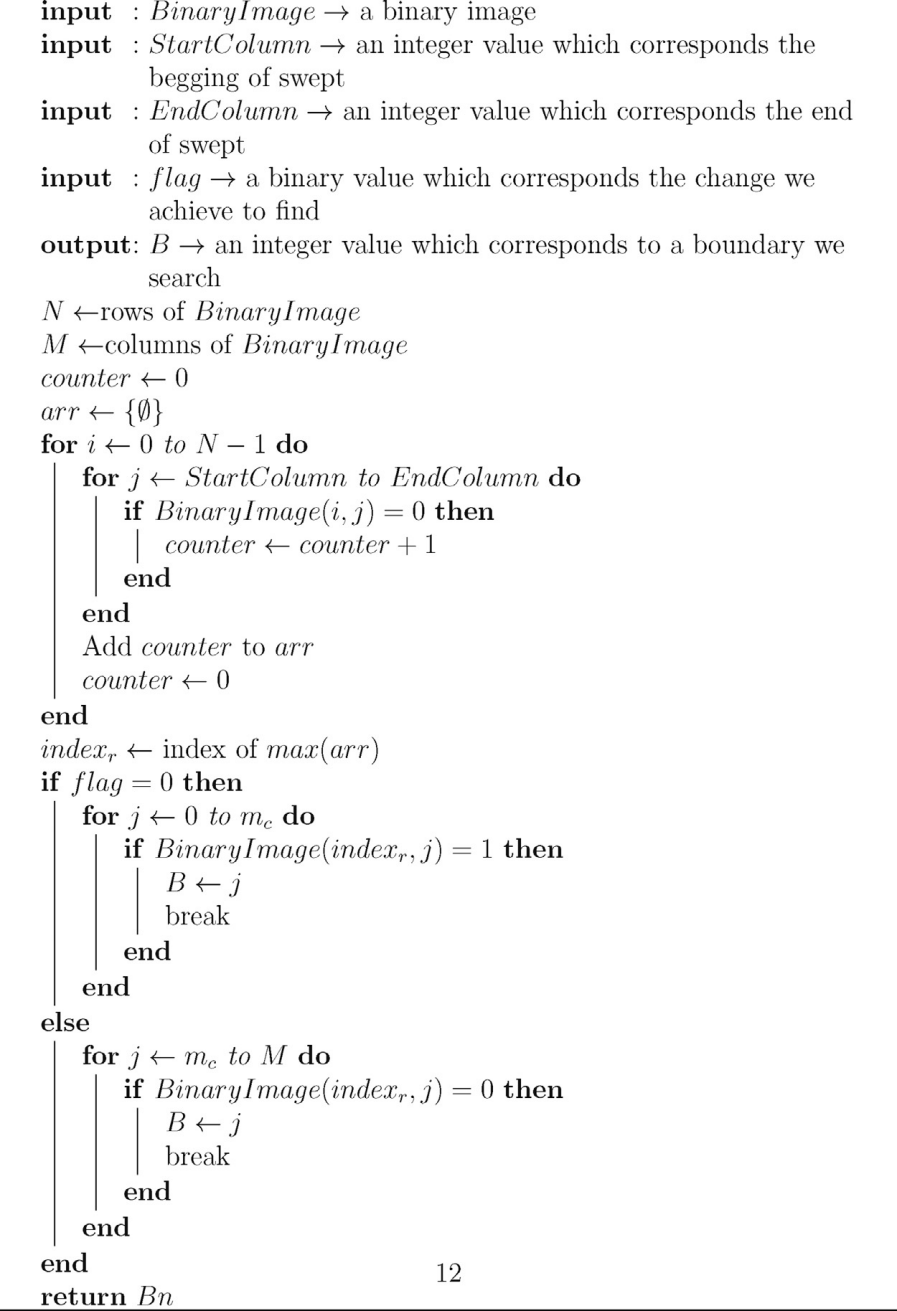# Method to extract an enhanced cervical vertebrae area from a digital X-ray image

**DOI:** 10.1016/j.mex.2018.06.009

**Published:** 2018-06-19

**Authors:** E. Martínez-Sandoval, Miguel E. Martínez-Rosas, Humberto Cervantes de Ávila, Manuel Moisés Miranda Velasco, M.R. González-Márquez

**Affiliations:** Universidad Autónoma de Baja California, Ensenada, México

**Keywords:** Cervical vertebrae, Digital X-ray image, Threshold selection, Image-enhancing, Dynamic range

## Abstract

Combination of digital X-ray with image processing techniques has the potential to extract useful information for healthcare professionals (physicians). From all the information that can be extracted from X-ray images, information concerning the human cervical vertebrae is relevant for the medical area. Therefore, in this work we present a simple enhanced region of interest (ROI) selection tool to select automatically the region that contains most of the information concerning to cervical vertebrae. The ROI-selection method reduces the size of a lateral or frontal digital X-ray by 30–60% without losing significance in the resulting image. This is achieved by an adjustment of dimensions in the image while the cervical area is preserved. Moreover, the visual quality is improved by performing a contrast enhancement in the region of interest.

• Automatic threshold selection is computationally more efficient than traditional image segmentation techniques.
• Reduce size in comparison with original image (enhancing ROI).
• Independence of depth gray scale space.

## Graphical abstract

**Figure:**
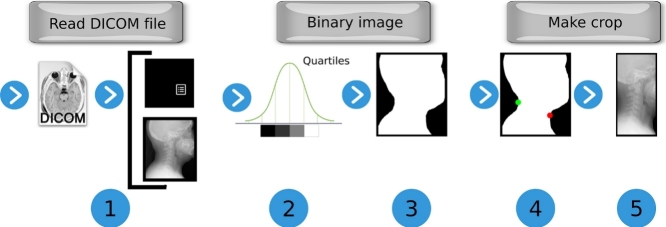

## Method details

A gray-level image is required as input for the method. Then, a dynamic threshold routine is implemented in order to obtain a binary image. The image must include uniform closed objects in order to extract their features (centers and dimensions). From extracted data, the largest object was identified in the image, which correspond to the patient's silhouette. This selection is a crucial step in the method.

At this point, the algorithm performs a scan all over the image matrix in order to identify the coordinates of the object-background boundary. The aim of this stage is to define the narrowest area of the object, which corresponds to the neck of the patient (Region of Interest). Obtained data is used to identify two coordinates for the narrowest area in the closed object. Those coordinates are used to define two virtual boundaries corresponding to the horizontal limits of the ROI. In the resulting area, a size reduction between 30% and 60% is achieved, while the cervical vertebrae region is preserved. The output of the process is an image section which size-to-useful information ratio is improved regarding the original image. The output image is focused in the cervical vertebrae area and its quality remains as in the original image. However, due to the reduction of the gray-level dynamic range in the output image, it is performed a contrast enhancement based on histogram equalization, in order to use all the available range.

The following pseudocode describes the required steps to find out the right and left limits from the image, a matrix scan is performed over the binary image in order to determine the narrowest useful area in the ROI image.

Algorithm 1 Detection of boundaries in reduction process.

**Table:** 

**input**: *B*(*i*, *j*) → a binary image
**input**: *m*_*c*_ → an integer value which corresponds to the central column
**output**: *Bn* → an array with two values: *Bn*_0_ left boundary and *Bn*_1_ right boundary
*M* ⟵ columns of *B*
*Bn*_0_ ⟵ 0
*Bn*_1_ ⟵ *m*_*c*_ − 1
*Bn*_0_ ⟵ *countMaxZeros*(*B*, 0, *m*_*c*_, 0)
*Bn*_1_ ⟵ *countMaxZeros*(*B*, *mc*, *M* − 1, 1)
**return** [*Bn*]

Description: *countMaxZeros(BinaryImage,StartColumn,EndColumn,flag):* This function sweeps the input image *BinaryImage* (binary image preferably) from *StartColumn* (an integer index value) to *EndColumn* (another integer index value), locating the row with the maximum number of zeros and extract the column where there is a change from 0 to 1 or vice versa (If *flag value is 0, the change we search is from 0 to 1, if it is 1, the change searched will be from 1 to* 0). The process is shown in additional section (Algorithm 2).

## Stages in the algorithm

1. Pre-processing image data
  • Digital images used to test the method comply with Digital Imaging and Communication in Medicine (DICOM) protocol, so it is required to remove metadata from the image file. In order to accomplish the mentioned requirement *Medpy* and *Pydicom* python packages were used.
2. Image complement
  • Negative films plate are used in the typical X-ray images, so that the densest objects are observed as white. The opposite happens in digital X-ray, where the sensors that replaced the plate receive less intensity due to absorption in dense objects and are observed darker. For interpretation reason it is convenient to use complements of the images [4], [1].
  • Gray level ranges vary from image to image, which increase the complexity for data manipulation as mentioned in [3]. On the other hand, when handling the complement image, histogram origin for all of the images is 0, so we are able to use this as reference.
    $$\begin{array}{c} C(i,j)=\max(A)-A(i,j)\\ \text{where}\;A=\text{Original image},\\ C=\text{Complement image} \end{array}$$
3. Image histogram
  • The histogram represents the distribution of intensity levels points on the image.
4. Histogram is divided into quartiles.
  • By analyzing the diversity of histograms from the worst image to the best image, a dynamic threshold value is required in order to suit each image range.
  • First quartile (0–25%) is used to define the dynamic threshold value.
5. The image is binarized using the dynamic threshold value found on the histogram by the quartile 1 (0–25%) in every image.
  • The aim of binarizing an image using a dynamic threshold value is to generate clear silhouettes, which are interpreted as a closed object.
6. Extracting object features.
  • The *findContours* function from *OpenCV* is used to obtain image parameters (center, width, height) that allow us to calculate the object proportions.
  • The object with the largest area (silhouette) is selected, this process removes unwanted objects.
7. A virtual boundary is drawn on the central coordinate and the object is divided into two parts.
  • Taking as reference the center coordinate of the object with the largest area, the image is divided vertically with a virtual boundary.
8. Location of coordinates for the narrower area of the object.
  • The matrix is swept in order to find the coordinate with the highest number of zeros per row. In both sections of the image.
  • The image matrix is analyzed from the left boundary to the virtual boundary in order to locate the coordinate 1.
  • The image matrix is analyzed from the virtual boundary to the right boundary in order to locate the coordinate 2.
9. The coordinates of the resulting image are shifted taking as reference the coordinates of the original image.
  • Points *i* and *i*′ are set as upper and lower boundaries respectively. In order to preserve visually useful information, these values were fixed to the original values of the image, which were obtained during the reading of the image.
  • The points *j* and *j*′ are set as left and right boundaries, respectively. This can be interpreted as the image from left to right.
  • (*i*, *j*) and (*i*′, *j*′) coordinates correspond to the boundaries where the information of interest is located.
10. A sub-image is generated with an image that properly fits the cervical vertebrae area.
11. A new histogram corresponding to the obtained image is generated.
12. A contrast enhancement is performed through histogram equalization in the new image section in order to use all available gray level range [2].
13. The new image section with an enhanced contrast is saved for further usage.

The process is described graphically in 5 stages.

1. The image information and metadata are initially separated from the original file (see Fig. 1a). The original image histogram and its accumulated (Fig. 1b and c, respectively) indicate the frequency distribution of the gray-level values of the image. This is helpful to locate the frequency of specific values and the range of gray-level values contained in the image.

**Fig. 1:**
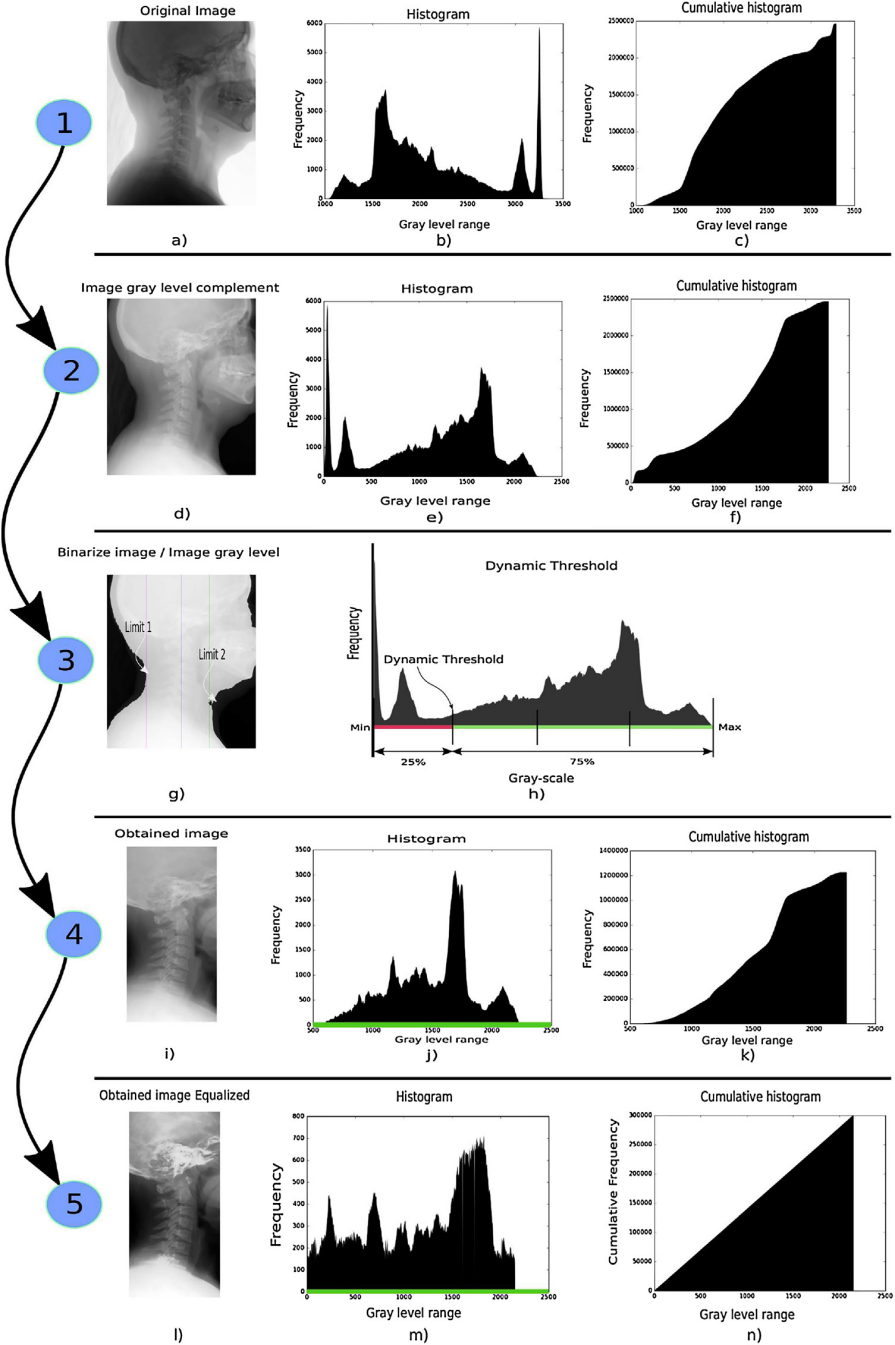
Full graphical process.
2. The information is adjusted to be interpreted as a typical X-ray image, the objects are represented in white tones and background in dark tones (see Fig. 1d). Typically, image histograms at the origin of gray-level values range correspond to dark tones while the white tones correspond to higher values (see Fig. 1e). In addition, the complimentary information of the image is presented by the cumulative histogram (see Fig. 1f).
3. A dynamic threshold segmentation based on the quartile 1 of the histogram is applied (see Fig. 1h), which results in a binary image mask to find two coordinates that will be used as boundaries (see Fig. 1g).
4. The original complement image (Fig. 1d) is resized according to the boundaries obtained from results of the previous stage in what is described as ROI. Such ROI represents 30–60% of the information regarding the original image (see Fig. 1i). The histogram of the new image, is quite different from the original one since it only contains information about the resulting ROI (see Fig. 1j).
  The cumulative histogram is smoother because most of the information within the image corresponds to bone-tissue, as a result there are no abrupt transitions (see Fig. 1k).
5. The contrast enhancement is done through the histogram equalization to use the entire dynamic range of the image (see Fig. 1i). The histogram of the enhanced image shows a change of shape due to the mentioned process (see Fig. 1m). Furthermore, the cumulative histogram illustrates a uniform distribution of gray-level values due to histogram equalization (see Fig. 1n).

Taking into account the results obtained, the method adequately reduces the size of the image while preserving the area of the cervical vertebrae of lateral or frontal digital X-ray images. In addition, the contrast enhancement due to histogram equalization can be easily noticed, this feature can be useful for specialized diagnoses. The proposed method can be implemented as a pre-processing stage for a deeper analysis.

## Additional

**Fig. 2:**
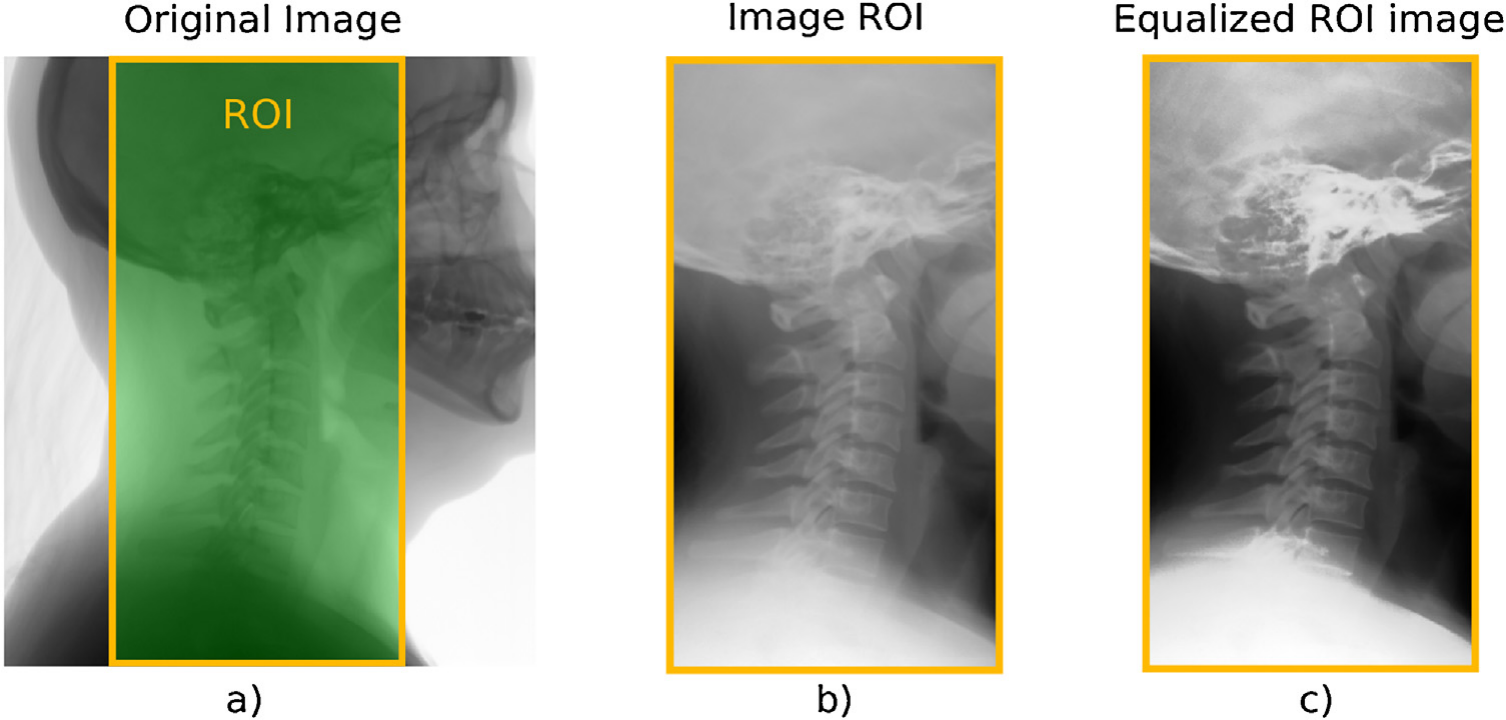
(a) ROI section, (b) resized-image, and (c) enhanced-image.

Fig. 2 shows the sequential partial results obtained by applying the proposed method on a digital X-ray image. Sub-image (a) shows the original image with region of interest highlighted in green. Sub-image (b) shows the reduced-size obtained image. Finally, the sub-image (c) shows the output enhanced image resulting from the method.

**Fig. 3:**
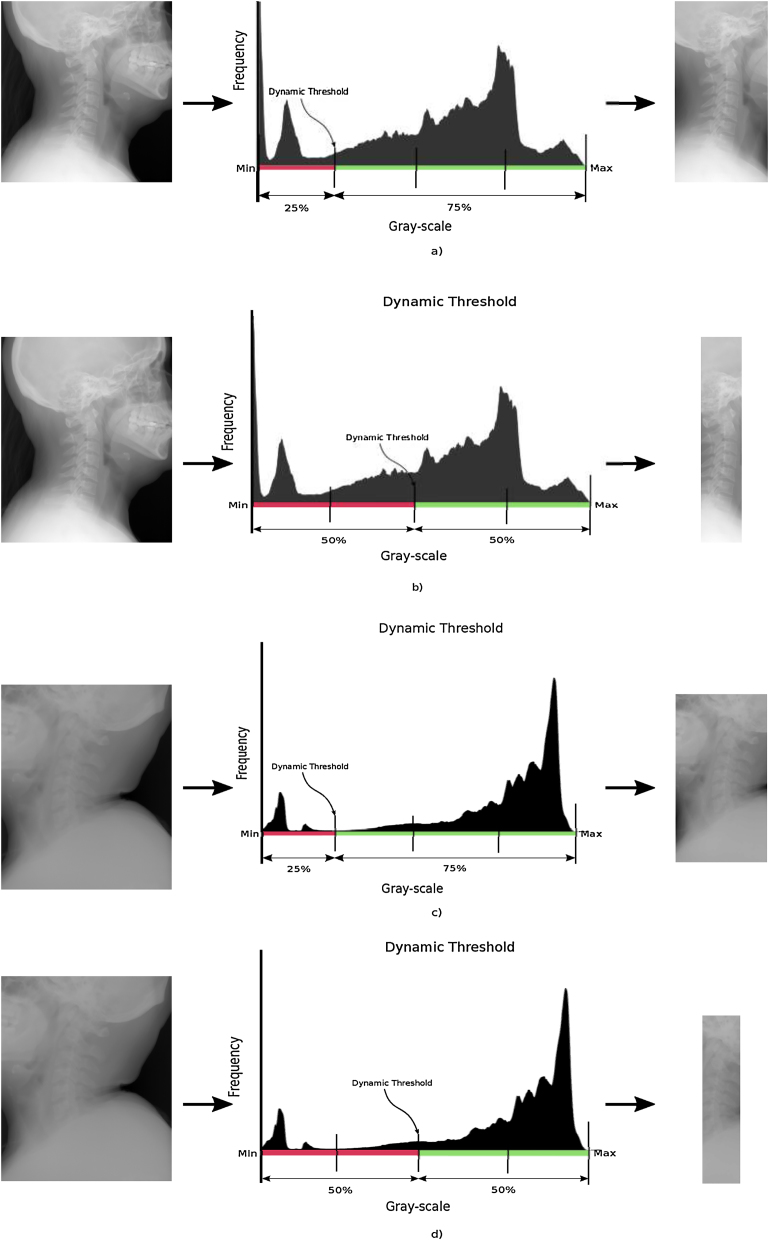
Dynamic threshold.

Fig. 3 shows resulting images from: proper threshold selection (a, c) and unsuitable threshold selection (b, d). This figure shows that selecting a threshold value higher than the limit of quartile 1 produces a reduced area which overlaps with the cervical vertebrae ROI and then causes loss of useful information.

**Fig. 4:**
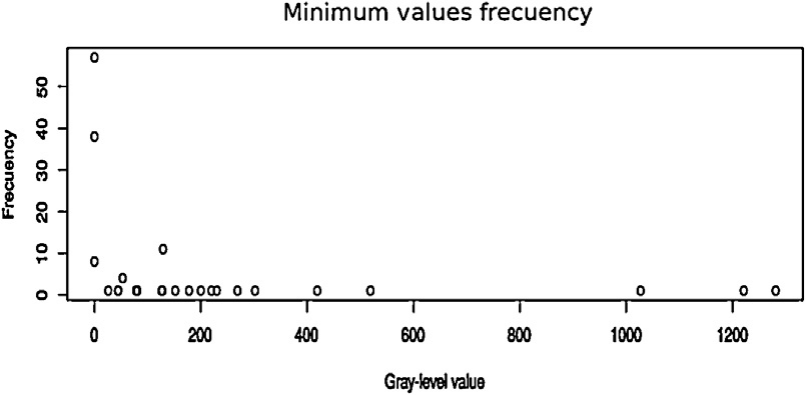
Scatter of lower limits of original images ranges.

**Fig. 5:**
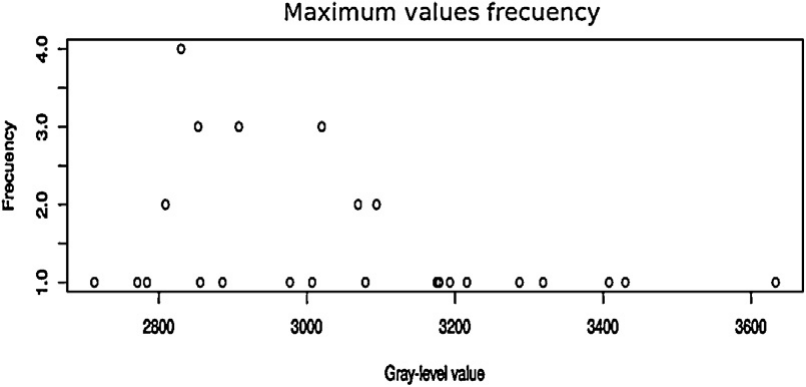
Scatter of lower limits of original images ranges.

**Fig. 6:**
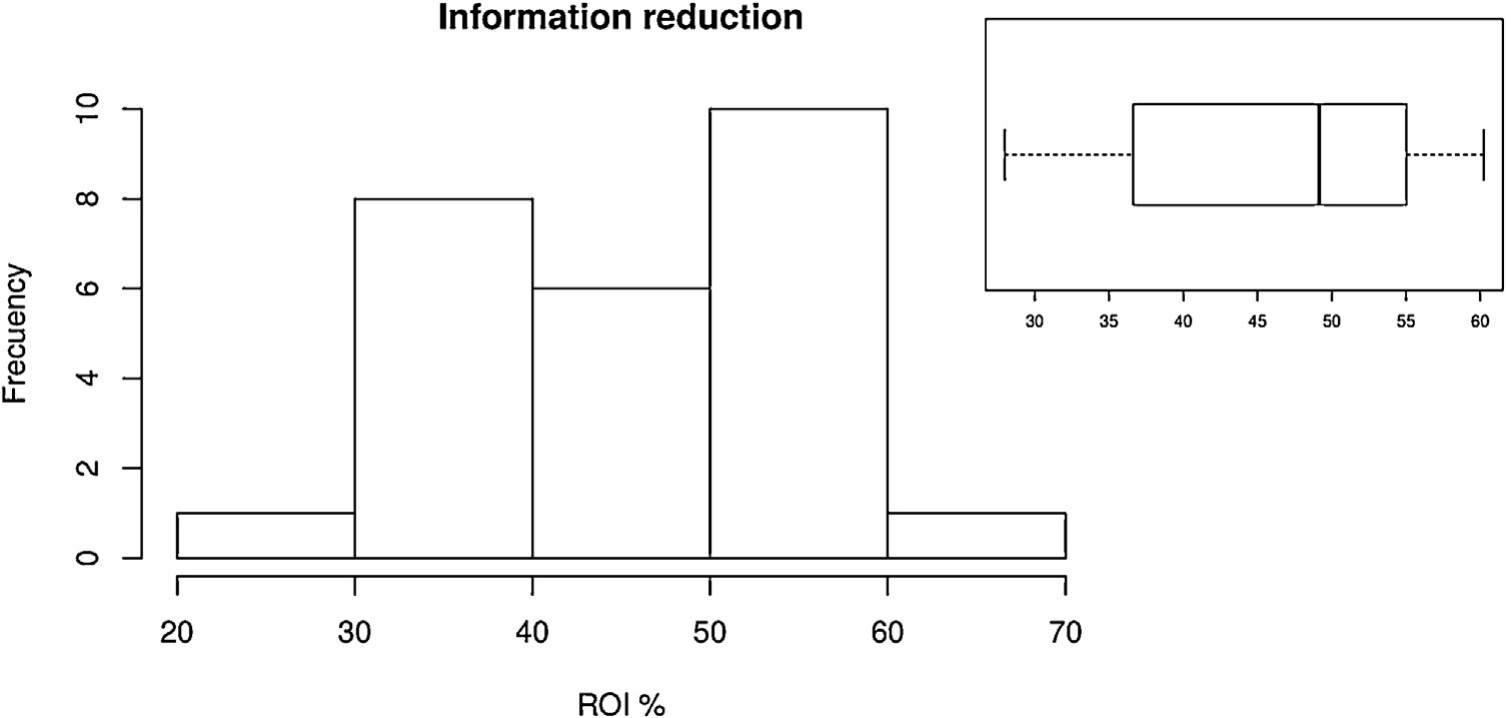
Reduction percent regarding the original image.

Fig. 4, Fig. 5 show the scatter of minimum and maximum gray-level values from the dynamic range corresponding to the set of original images respectively. In both cases it is easily observed that the gray-level values are located on the darkest region which approximately correspond to the lowest 10% of the full dynamic range in 16 bit gray-level scale (0–65535 gray-level values). Fig. 6 shows the percentage of the ROI distribution where an important information reduction can be observed.

Algorithm 2 Function *countMaxZeros*.

**Figure:**